# Feline Calicivirus Virulent Systemic Disease: Clinical Epidemiology, Analysis of Viral Isolates and In Vitro Efficacy of Novel Antivirals in Australian Outbreaks

**DOI:** 10.3390/v13102040

**Published:** 2021-10-09

**Authors:** Matteo Bordicchia, Tulio Machado Fumian, Kate Van Brussel, Alice G. Russo, Maura Carrai, Shi-Jia Le, Patricia A. Pesavento, Edward C. Holmes, Vito Martella, Peter White, Julia A. Beatty, Mang Shi, Vanessa R. Barrs

**Affiliations:** 1 Faculty of Science, Sydney School of Veterinary Science, The University of Sydney, Sydney, NSW 2006, Australia; matteo.bordicchia@sydney.edu.au (M.B.); kate.vanbrussel@sydney.edu.au (K.V.B.); julia.beatty@cityu.edu.hk (J.A.B.); 2 Faculty of Science, School of Biotechnology and Biomolecular Sciences, University of New South Wales, Sydney, NSW 2052, Australia; tuliomf@ioc.fiocruz.br (T.M.F.); a.russo@nsw.edu.au (A.G.R.); p.white@unsw.edu.au (P.W.); 3 Laboratory of Comparative and Environmental Virology, Oswaldo Cruz Institute, Oswaldo Cruz Foundation, Fiocruz 4365, Brazil; 4 Marie Bashir Institute for Infectious Diseases and Biosecurity, School of Life and Environmental Sciences and School of Medical Sciences, University of Sydney, Sydney, NSW 2006, Australia; edward.holmes@sydney.edu.au; 5 Department of Veterinary Clinical Sciences, Jockey Club College of Veterinary Medicine and Life Sciences, Centre for Companion Animal Health, City University of Hong Kong, Kowloon Tong, Hong Kong 999077, China; mcarrai@cityu.edu.hk; 6 School of Medicine, Sun Yat-sen University Guangzhou East Campus, Panyu, Guangzhou 510080, China; leshij@mail2.sysu.edu.cn (S.-J.L.); shim23@mail.sysu.edu.cn (M.S.); 7 Department of Pathology, Microbiology and Immunology, UC Davis School of Veterinary Medicine, 1044 Haring Hall, 1 Shields Avenue, Davis, CA 95616, USA; papesavento@ucdavis.edu; 8 Department of Veterinary Medicine, University of Aldo Moro of Bari, 70010 Valenzano, Italy; vito.martella@uniba.it

**Keywords:** Caliciviridae, *Vesivirus*, nitazoxanide, 2′-C-methylcytidine, NITD-008

## Abstract

Feline calicivirus (FCV) causes upper respiratory tract disease (URTD) and sporadic outbreaks of virulent systemic disease (FCV-VSD). The basis for the increased pathogenicity of FCV-VSD viruses is incompletely understood, and antivirals for FCV-VSD have yet to be developed. We investigated the clinicoepidemiology and viral features of three FCV-VSD outbreaks in Australia and evaluated the in vitro efficacy of nitazoxanide (NTZ), 2′-C-methylcytidine (2CMC) and NITD-008 against FCV-VSD viruses. Overall mortality among 23 cases of FCV-VSD was 39%. Metagenomic sequencing identified five genetically distinct FCV lineages within the three outbreaks, all seemingly evolving in situ in Australia. Notably, no mutations that clearly distinguished FCV-URTD from FCV-VSD phenotypes were identified. One FCV-URTD strain likely originated from a recombination event. Analysis of seven amino-acid residues from the hypervariable E region of the capsid in the cultured viruses did not support the contention that properties of these residues can reliably differentiate between the two pathotypes. On plaque reduction assays, dose–response inhibition of FCV-VSD was obtained with all antivirals at low micromolar concentrations; NTZ EC_50_, 0.4–0.6 µM, TI = 21; 2CMC EC_50_, 2.7–5.3 µM, TI > 18; NITD-008, 0.5 to 0.9 µM, TI > 111. Investigation of these antivirals for the treatment of FCV-VSD is warranted.

## 1. Introduction

Feline calicivirus (FCV) is a single-stranded, positive-sense, non-enveloped RNA virus in the genus *Vesivirus,* family Caliciviridae. The 7.5 kb FCV genome contains three overlapping open reading frames (ORFs) that encode non-structural proteins (ORF 1), the VP1 major capsid protein (ORF 2) and the VP2 minor capsid protein (ORF 3). The 5’ end of the genome is linked to the viral protein genome (VPg) and the 3’ end is polyadenylated [1,2,3,4]. Genetic variants of FCV are generated constantly through polymerase “errors” and recombination events. FCV is highly contagious and a common cause of acute upper respiratory tract disease (URTD) in cats, characterised by fever, oculonasal discharge, sneezing and oral ulceration. Polyarthritis and mucocutaneous ulcers are less common features [5]. The virus is spread directly and by contact with contaminated fomites. Young cats and kittens in multicat environments are most susceptible to FCV-URTD, but most recover with supportive care. Vaccination against FCV, which is routine, prevents or limits disease.

FCV also causes clinically distinct outbreaks of virulent systemic disease (FCV-VSD), especially in adults, with mortality rates of up to 79% [6,7,8,9,10,11,12,13]. FCV-VSD is characterised by systemic vasculitis and severe epithelial necrosis resulting in oedema of the head and limbs, multifocal ulceration of the skin and footpads, jaundice and pneumonia. Concurrent FCV-URTD is usually present [7,8,12]. As vaccination does not fully protect against FCV-VSD, there is interest in developing antivirals for use in the face of outbreaks. Three antiviral drugs, including two nucleoside analogues (2′-C-methylcytidine and NITD-008) and a thiazolide anti-infective agent (nitazoxanide), have been shown to have efficacy against the FCV-F9 prototype vaccine strain, but they have not yet been tested against viruses isolated from FCV-VSD outbreaks [14,15].

Attempts to define distinguishing molecular signatures of the highly pathogenic viruses isolated from cases of FCV-VSD have thus far been unsuccessful [10,11,16]. However, in a recent study, it was proposed that the majority of FCV-URTD and FCV-VSD viruses, or pathotypes, could be distinguished based on the physical and chemical properties of seven key amino acids in the hypervariable E region of VP1 (residues 426–521) involved in receptor binding and cell entry [17]. Specifically, a multiple correspondence analysis (MCA) suggested that these properties related to residues 438 (non-polar with an aliphatic chain), residue 440 (not small), residue 448 (polar with positive charge), residue 452 (not small), residue 455 (not negative), residue 465 (polar) and residue 492 (small) [17]. Whether this method is reliable for differentiation of FCV-VSD pathotypes requires further testing using a larger number of isolates.

Between 2015 and 2018, three separate nosocomial outbreaks of suspected FCV-VSD were reported in New South Wales (NSW), Queensland (QLD) and the Australian Capital Territory (ACT), in Australia. To the best of our knowledge, FCV-VSD has not been reported previously in the southern hemisphere. Herein, we aimed to provide a clinical and epidemiological description of the outbreaks, to investigate viral isolates for FCV-VSD pathotype-specific properties and to determine the in vitro efficacy of nitazoxanide (NTZ), 2′-C-methylcytidine (2CMC) and NITD-008 against FCV-VSD viruses. These antiviral agents were selected for testing against FCV-VSD strains based on the results of previous investigations [14,15]. In one study, from among 15 antiviral agents evaluated, NTZ and 2CMC were identified as potent inhibitors of replication of the F9 vaccine strain in vitro, with EC_50_ values in the low micromolar range [14]. Subsequently, NITD-008 was evaluated against three calicivirus model systems (FCV, murine norovirus and the human norovirus (Norwalk) replicon) and found to be a potent inhibitor of replication of all three caliciviruses in vitro, with low toxicity and EC_50_ values of <1 µM [15].

## 2. Materials and Methods

### 2.1. Case Data and Definitions

Outbreaks of FCV-VSD were reported at a single first-opinion veterinary hospital in Sydney, NSW, in 2015 (Outbreak 1); Ipswich, QLD, in 2017 (Outbreak 2) and Canberra, ACT, in 2018 (Outbreak 3). Data obtained from medical records of patients presenting with signs of FCV-VSD and/or FCV-URTD included age, breed, sex, neuter status, FCV vaccination history, clinical signs, duration of illness, treatments prescribed and outcome. Disease phenotype was assigned as either (a) FCV-URTD defined as the presence of ≥2 of the following clinical signs: fever (rectal temperature > 39.3 °C), ocular or nasal discharge, conjunctivitis, oral or mucocutaneous ulcers or lameness, or (b) FCV-VSD defined as ≥1 signs of FCV-URTD and ≥1 of the following: limb or facial oedema, jaundice, skin ulceration or death [5]. Vaccination status was classified as complete (documented completion of a primary FCV vaccination course and, where a year or more had elapsed, revaccination at least once within the preceding three years), incomplete (cats <16 weeks of age that had received one or more vaccinations against FCV or cats that had not been revaccinated for >1 year after the primary vaccination course), unvaccinated (never vaccinated) or unknown vaccination status.

### 2.2. Sample Collection

Tissue samples were available from two cases that died during Outbreak 1. Cadavers of these cats were stored at −20 °C before transport to our laboratory for sample collection. Samples of liver, lung, lymph node and ulcerated skin were collected and stored at −80 °C until processing for viral culture. Oropharyngeal and conjunctival swabs were collected from cats showing clinical signs associated with the different pathotypes from Outbreak 2 (*n* = 9) and Outbreak 3 (*n* = 5). In addition, swabs were collected from a cat (QLD_13 KL) showing URTD signs during Outbreak 2 from a nearby veterinary hospital. Swabs were also collected from an asymptomatic cat (ACT_3) that had presented for a routine dental procedure during Outbreak 3. Swab tips were placed in 0.5 mL of viral transport medium (Dulbecco’s modified Eagle’s medium, with 1000 mg L^−1^ glucose, L-glutamine and sodium bicarbonate + 10% foetal bovine serum) and shipped at 4 °C for viral culture.

### 2.3. Virus Isolation

FCV was isolated from swab and tissue specimens on confluent Crandell–Rees feline kidney (CRFK) cells at 37 °C in 5% CO_2_ in Dulbecco’s modified Eagle’s medium, with 1000 mg L^−1^ glucose, L-glutamine and sodium bicarbonate + 10% foetal bovine serum + streptomycin 5000 U/mL and amphotericin B 25 μg/mL [9]. One mock infected monolayer was incubated in parallel with each batch of samples and was considered negative if CPEs were absent after 5 days. Samples were considered positive for FCV if they developed characteristic cytopathic effects (CPEs) within 12–72 h post infection. Viruses were harvested, stored and processed separately to avoid cross-contamination.

### 2.4. Histopathology and Immunohistochemistry (IHC)

One case from Outbreak 2 that succumbed with clinical signs of FCV-VSD was submitted for post-mortem examination, including histopathology. Formalin-fixed paraffin-embedded liver tissue was immunohistochemically stained using the monoclonal anti-FCV clone S1–8 provided by Customs Monoclonals Intl., Sacramento, CA, USA, as previously described [13].

### 2.5. RNA Extraction RT-PCR

To confirm isolation of FCV, RNA was extracted from positive cultures using the RNAeasy Mini Kit (Qiagen, Hilden, Germany), according to the manufacturer’s guidelines. Reverse transcription was performed using 200 ng random hexamers (SuperScript^®^ III One-Step RT-PCR System, Thermo Fisher Scientific Inc., MA, USA). FCV capsid amplification by PCR was carried out as described [18]. Reverse transcription was carried out at 52 °C for 10 min, followed by initial denaturation at 95 °C for 5 min, then 35 cycles of denaturation at 95 °C for 1 min, annealing at 56–57 °C for 15 s, extension at 72 °C for 10 s, and a final extension at 72 °C for 5 min. The identity of PCR products migrating at the expected size on gel electrophoresis was confirmed by Sanger sequencing (Macrogen sGenome Centre, Seoul, Korea). Next-generation sequencing (NGS) libraries were prepared from viral cultures of selected positive cases from each outbreak after RNA extraction, using the TruSeq RNA library preparation kit (Illumina, San Diego, CA, USA), and cytoplasmic ribosomal RNA was depleted using a Ribo-Zero Gold rRNA removal kit (Human/Mouse/Rat) (Illumina, USA). RNA sequencing of 100 bp paired-end libraries was performed on an Illumina NovaSeq 6000 platform.

### 2.6. Bioinformatic and Phylogenetic Analyses

The resulting sequencing reads were assembled, followed by the identification of full-length FCV genomes. The reads were assembled using Megahit [19], and the assembled sequences were annotated using diamond Blastx against the entire non-redundant (nr) database on GenBank. Contigs associated with FCV genomes were then identified, and complete virus genomes were obtained after mapping virus reads against the virus contigs. The assembled genomes were then compared with (i) complete or near-complete FCV genomes as well as (ii) partial VP1 gene sequences downloaded from GenBank, which were more representative of FCV diversity. Both datasets contained previously identified virulent strains of FCV. Phylogenetic trees were estimated based on both datasets using the maximum-likelihood algorithm implemented in the PhyML (version 3.0) [20], employing a general-time reversible (GTR) substitution model and subtree pruning and regrafting (SPR) branch-swapping algorithm. Support for the phylogeny was evaluated using an approximate likelihood ratio test (aLRT) with the Shimodaira–Hasegawa-like procedure.

The inferred amino-acid (aa) sequences of the capsid protein of Australian FCV viruses were aligned with reference FCV strains from cats with VSD or URTD as well as with enteric strains of FCV from cats with diarrhoea [21]. The specific physical and chemical properties of the seven residues previously identified as linked to the VSD pathotype were assigned binary outcomes (0 = no, 1 = yes) and compared for aa 438 (polar, aliphatic), aa 440 (small), aa 448 (polar, charged, small, positive), aa 452 (small), aa 455 (negative, charged), aa 465 (hydrophobic, polar) and aa 492 (small) [17]. Multiple correspondence analysis based on the Burt matrix of these 13 categorical variables was performed in XLSTAT 2020 (Addinsoft Pty. Ltd., Paris, France) in a blind analysis where pathotype (VSD vs. non-VSD) was not included as a variable.

### 2.7. Recombination Analysis

To identify possible recombination events among these sequences, we collected 56 FCV full-length genomes including the Australian outbreak viruses and genomes available on GenBank. Sequences were aligned using MAFFT v 7.0 [22] and then analysed for recombination events using the SimPlot program 3.5.1 [23] with a 1000 bp window size and 10 bp step size. In SimPlot analyses, Australian strains were used as a query and compared against representative viral genomes and outgroup viral genome sequences. Recombination breakpoints were identified based on observation of a cross-over of similarity against two different strains. Parental strains for each potential recombinant were confirmed by reconstructing phylogenies based on each non-recombinant region separated by breakpoints.

### 2.8. Evaluation of Antivirals in Plaque-Reduction Assays

The *in vitro* efficacy of the antivirals nitazoxanide (NTZ; Sapphire Bioscience, NSW, Australia), 2′-C-methylcytidine (2CMC; Sigma-Aldrich, St. Louis, MO, USA) and NITD-008 (In Vitro Technologies, VIC, Australia) against two FCV viruses isolated from cases of severe FCV-VSD, and the F9 vaccine strain, was evaluated using plaque-reduction assays, as previously described [14]. Briefly, NTZ, 2CMC and NITD-008 were dissolved in 100% dimethyl sulphoxide (DMSO) and the stock solutions (10 mM) were aliquoted and stored at −20 °C. CRFK monolayers (8 × 10^5^ cells/well) in 6-well plates were inoculated with 80 plaque forming units (pfu) of FCV for 1 h at 37 °C, followed by the addition of semisolid agarose overlays containing different concentrations of each antiviral in triplicate. After 24 h incubation, cells were fixed and stained with crystal violet, and plaque numbers were determined for each treatment well. DMSO vehicle control was defined as having maximal viral infectivity. At least two independent experiments were performed for each treatment, with results presented as the mean with standard error of the mean (SEM). The therapeutic index was calculated from the half-maximal efficacy concentration (EC_50_) and half-maximal cytotoxic concentration in CRFK cells (CC_50_). The EC_50_ and CC_50_ were assessed as previously described [14]. The therapeutic index was then calculated (TI = CC_50_/EC_50_).

## 3. Results

### 3.1. Clinical and Epidemiological Descriptions of Outbreaks

In total, 23 cases of FCV-VSD were identified, including seven cases from Outbreak 1 (NSW_3 to NSW_9; Table 1), 12 from Outbreak 2 (QLD_1 to QLD_12; Table 2) and four from Outbreak 3 (ACT_4 to ACT_6 and ACT_9; Table 3). Patients ranged in age from 5 weeks to 8 years (median 12 months). In all, 22 of the 23 cases had been presented to the veterinary hospital involved in the outbreak for surgical procedures or consultations within the previous 14 days (*n* = 17) or were household contacts of such (*n* = 5; QLD_8, QLD_11, ACT_4, ACT_7, ACT_9; Table 2 and Table 3). In one case, the source of exposure was not identified (NSW_6, Table 1). The median time from exposure to the onset of clinical signs was 7 days (range 2 to 14 days). FCV vaccination status was known in 21 cases and was complete in 19 and incomplete in one, while one cat was unvaccinated. The overall mortality rate was 9/23 (39%) and was highest in Outbreak 1, 6/7 (86%), intermediate in Outbreak 2 (3/12, 25%) and zero in Outbreak 3 (0/4, 0%). Hospitalised patients were treated with supportive treatment, including intravenous fluids and analgesia. The duration of the outbreaks was 4 to 6 weeks with no spread beyond the hospital identified in each outbreak.

Index cases were identified in Outbreaks 1 and 3. Outbreak 1 occurred after two unrelated rescue cats (NSW_1 and NSW_2) were hospitalised with signs of FCV-URTD, including lameness (Table 1). Outbreak 3 occurred after a cat with signs of FCV-URTD, including lameness, was admitted to hospital shortly after being adopted from a shelter (ACT_1, Table 3). Its littermate, also had signs of FCV-URTD (ACT_2, Table 3). No index case was identified for Outbreak 2, but the hospital at the centre of the outbreak was noted to service a large on-site animal shelter.

### 3.2. Histology and Immunohistochemistry

A post-mortem examination was performed on case QLD_4 from Outbreak 2. Diagnostic oropharyngeal swabs submitted antemortem to a commercial laboratory tested positive for FCV on qPCR, and negative for FHV-1. At post-mortem, diffuse oedema of the head, including the conjunctivae, and generalised subcutaneous oedema of the body were noted. Low-volume serosanguinous pleural (60 mL), abdominal (8 mL) and pericardial (2 mL) effusions were present, and the tissues of the mesentery were icteric. On histopathology, a fibrinonecrotising interstitial pneumonia, diffuse piecemeal hepatic necrosis and palpebral epidermal necrosis with suppurative adnexal dermatitis were described. No bacteria were isolated on aerobic and anaerobic culture of the effusion. FCV antigen was not detected on immunohistochemistry of sections of liver.

### 3.3. Virus Isolation and Genome Assembly

Liver and lung samples (*n* = 5) from both cases sampled in Outbreak 1 were culture positive for FCV, showing CPEs within 24 h and testing positive on RT-PCR assays for FCV (Table 1). From Outbreaks 2 and 3, 8/9 and 5/5 cases, respectively, were positive for FCV with CPEs detected within 36 to 72 h (Table 2 and Table 3). RNA extracts from 15 virus cultures were submitted for WGS (Table 1, Table 2 and Table 3) and whole FCV genomes were assembled (Table 1, Table 2 and Table 3).

### 3.4. Metagenomic, Amino-Acid and Recombination Analyses

**Figure 1:**
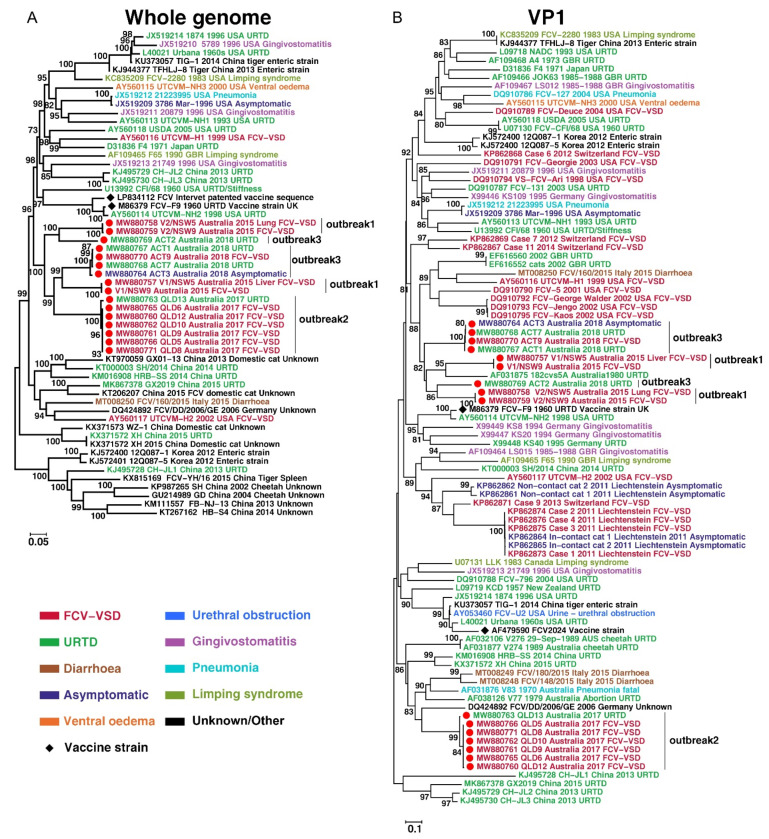
Evolutionary history of viruses discovered in this study. Maximum-likelihood trees were reconstructed based on (**A**) Whole-genome and (**B**) partial VP1 gene alignments, which included virus sequences obtained in this study (marked with solid red circles) as well as those obtained from the GenBank. The trees were mid-point rooted and bootstrap values of ≥70% are marked on the tree. Each sequence name contains the accession number, strain name, geographic location, host and isolation year, if available. Disease phenotype is colour coded.

From the 14 RNA library preparations, RNA sequencing produced median read counts per library after filtering of 126,025,385 (range 83,299,626 to 145,687,464) (Table S1). The median number of contigs per library was 32,127 (range 1860 to 891,879), and the median number of reads mapped per library was 3,213,060 (range 152,719 to 220,862,249). Fifteen whole FCV genomes were assembled from this data (GenBank accession numbers MW880757–MW880771, Figure 1, Table S1).

Phylogenetic analysis of the whole FCV genomes generated revealed that the three outbreaks were caused by five independent lineages (Figure 1). Outbreak 1 was associated with the co-circulation of two viruses of phylogenetically distinct lineages with a nucleotide identity of 80.25% (V1, in NSW_5 and NSW_9; V2 in NSW_5 and NSW_9). In Outbreak 2, FCV-VSD viruses were highly conserved genetically, with a nt identity of >99.84%. A virus isolated from a cat with URTD (QLD_13) from a different facility to that where Outbreak 2 was reported was more closely related to the Outbreak 2 viruses (98.85% nt identity) than to other Australian FCV viruses (Figure 1). In Outbreak 3, one FCV-VSD case (ACT_9) and two FCV-URTD cases (ACT_1 and 7) were identified that shared >99.73% nt genomic identity. Interestingly, a virus (strain ACT_3) cultured from an asymptomatic cat co-housed with an FCV-VSD case was most closely related to the outbreak virus (98.69% nt identity, Figure 1). Nevertheless, an FCV-URTD virus (strain ACT_2) from a cat co-housed with the index case (ACT_1, Table 3) and recently adopted from a shelter was from a different lineage than the FCV-VSD viruses from Outbreak 3 (67.16% nt identity). Instead, it clustered with strains NSW_5 and 9 from Outbreak 1, but with only a distant relationship (82.67% nt identity).

Collectively, viruses identified from the three outbreaks formed a monophyletic cluster (i.e., shared a common ancestor) that was distinguished from previously described viruses based on the full-genome phylogeny (Figure 1). However, the phylogeny based on the VP1 gene alone suggested two separate clusters: (i) one was associated with Outbreak 2 virus sequences alone and distantly grouped with viruses from China, Germany and Australia, and (ii) a second cluster was associated with the remainder of the outbreaks studied here as well as an Australian virus strain (182cvs5A) identified from a cat with URTD in 1980. Of note, we did not observe any clustering of viruses from cats with different clinical presentations (VSD, URTD) or from asymptomatic cats in the phylogeny. Indeed, it was striking that even among highly identical sequences (>98.65% nucleotide identity), namely ACT_1, ACT_3, ACT_7 and ACT_9, different presentations (URTD, VSD, asymptomatic) were observed. These results suggest that there is no clear association between disease severity and virus genetic background, although this needs to be assessed with more data.

**Figure 2:**
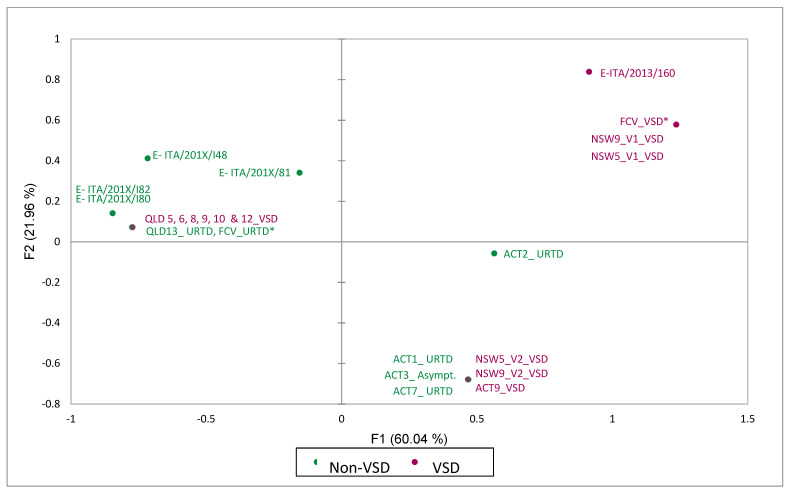
Multiple correspondence analysis (MCA) graph depicting the results of MCA of 13 physical and chemical categorical variables of 7 amino-acid residues of the hypervariable E region of VSD and non-VSD FCV strains from Table 4. * Most common amino-acid configuration of FCV_VSD and FCV_URTD reference strains (see Table 4); E-ITA, enteric FCV strains (see Table 4).

On residue mapping of the hypervariable region E of FCVs from Outbreak 1, one sequence type (NSW5_V1 and NSW9_V1) exhibited the proposed VSD-like pattern [17], whilst the other sequence type (NSW5_V2 and NSW5_V2) showed an intermediate configuration between the proposed pattern described for VSD and respiratory pathotypes (Table 4 and Figure 2). The FCV sequences from Outbreak 2 (QLD) had a residue pattern described by Brunet et al. [17] to be characteristic of the respiratory pathotype, while viruses from Outbreak 3 (ACT) had an intermediate configuration between the pattern described for VSD and respiratory pathotypes (Table 4, grey-shaded cells, and Figure 2).

**Figure 3:**
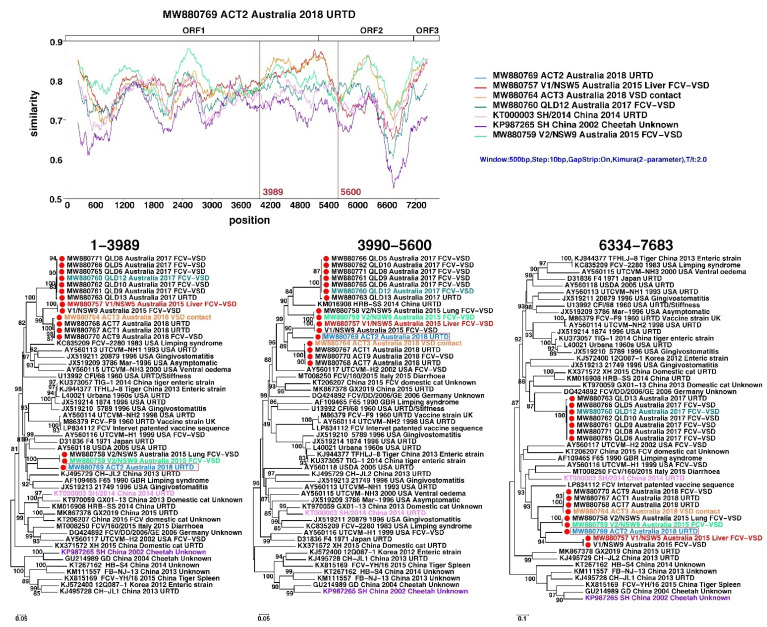
Recombination analyses of Australian FCV strains identified in this study. The top panel shows similarity comparisons of ACT2 against representative FCV strains across the entire genome using a sliding window (window size: 500 bp, step size: 10 bp). The potential recombination breakpoints are shown as red vertical lines. The bottom panel shows phylogenetic trees based on each non-recombinant region separated by recombination breakpoints. The strain names are labelled with different colours to mark the representative strains used in SimPlot analyses, including the parental groups for the recombinants.

The Australian strains identified in this study also contained multiple signals for recombination, although no close parental strains were identified. Based on similarity plots and phylogenetic analyses, the strongest recombination signal was observed in strain “ACT_2”: in 1–3989 bp of the genome alignment it clustered with two viruses from Outbreak 1 (V2/NSW_5 and 9), whereas for the rest of the genome it was with the other two viruses from Outbreak 1 (V1/NSW5 and 9) group (Figure 3). Other groups such as the one containing Outbreak 2 strains (QLD_5–6, 9–10 and 12–13) were also subject to alternative grouping in the phylogenetic analyses, although no close parental strains could be identified (Figure 3).

### 3.5. Antiviral Compound Efficacy

**Figure 4:**
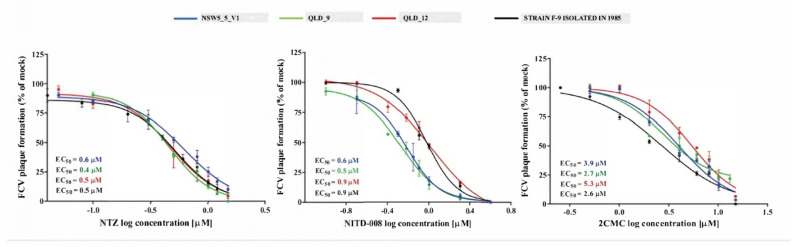
The antiviral activity of nitazoxanide (NTZ), NITD-008 and 2′-C-methylcytidine (2CMC) against four FCV strains in cell culture. The EC_50_ values of the three compounds against each of FCV strain were calculated by fitting the dose–response curves from a plaque reduction assay. Triplicate values from at least two independent experiments are presented, and the mean ± SEM are shown.

The EC_50_ values obtained for NTZ, 2CMC and NITD-008 tested against three FCV-VSD viruses from two of the outbreaks (NSW5_V1, QLD_9, QLD_12; Table 2 and Table 3), and from the F9 vaccine virus varied from 0.4 to 0.6 µM (0.1 to 0.2 µg/mL), 2.6 to 5.3 µM and 0.5 to 0.9 µM, respectively (Figure 4). NTZ showed a half-maximal cytotoxic concentration (CC_50_) value of 12.7 µM, whilst 2CMC and NITD-008 demonstrated CC_50_ values of >100 µM [14,15]. Therefore, using the higher EC_50_ values obtained for each compound, the therapeutic index (TI = CC50/EC50) values determined for NTZ, 2CMC and NITD-008 were 21, >18 and >111, respectively (Figure 4).

## 4. Discussion

This study documents the first epizootics of virulent systemic disease caused by FCV in Australia, although the association of FCVs with non-epizootic, atypical signs such as sudden death [24,25], jaundice [26] or severe ulcerative swelling of the footpads [27] was first described in Australia more than 20 years before FCV-VSD was reported in the U.S. [6]. Many features of the FCV-VSD outbreaks reported here resemble those reported in the U.S. [6,7,13,28], Europe [8,29] and the UK [30], including their nosocomial nature, the spectrum of clinical signs, outbreak duration of 4 to 6 weeks, absence of community transmission outside the affected hospital and, with 76% of affected cats being completely vaccinated, a non-protective effect of vaccination [6,8,28]. We also found no association between vaccination status and mortality. In Outbreak 1 in NSW, the only outbreak that involved unvaccinated cats, the proportion of unvaccinated or incompletely vaccinated cats that died was the same as that for vaccinated cats. Overall, the median time from exposure to the onset of clinical signs was longer (7 days) than in previous reports (4 to 4.5 days) [28,29]. However, the range was similar (from 1 to 14 days), supporting a minimum of 14 days quarantine for cats exposed during an outbreak.

The epidemiological and phylogenetic results of our study provide further evidence that any virus mutations responsible for the VSD phenotype arise de novo within multicat environments, in which persistent FCV infections are common and multiple FCV strains are circulating [7,28,30,31]. The strongest support comes from Outbreak 1, where the veterinary hospital routinely accepted unvaccinated rescued kittens and cats for rehoming that were subsequently housed together, with frequent mixing of kittens from different litters. The index cases, two unrelated 6-week-old kittens (NSW_1 and 2, Table 1) that developed an acute febrile illness and lameness one week before the FCV-VSD outbreak had been in the hospital from 2 weeks of age. Two other 6-week-old kittens that succumbed to FCV-VSD had been born in the hospital.

Notably, the viruses in each of the outbreaks were genetically distinct, did not cluster phylogenetically with other FCV-VSD viruses from previous outbreaks (Figure 1) and had no defining identifiable genomic mutations. This suggests extended in situ evolution in Australia of the viruses responsible for these outbreaks. Our study also confirmed the presence of high genetic diversity among FCV-VSD outbreak strains, which were present in multiple different clades, as has been previously described in other locations [28,31,32]. In general, only viruses sharing immediate temporal or spatial links, clustered together.

Analysis of amino acids from the hypervariable E region of the capsid in the cultured viruses did not support the hypothesis that the properties of the seven specific residues can reliably differentiate respiratory from virulent FCV pathotypes [17]. Indeed, the properties expected to be predictive of a virulent pathotype were present in all seven residues from only two viruses isolated from liver tissue of two FCV-VSD cases in Outbreak 1. A second FCV-virus, isolated from the lungs of the same cats, had these predicted properties in four of the seven residues. It is not possible to determine whether one or both strains were responsible for the observed FCV-VSD phenotype. We cannot exclude the possibility of cross-contamination during sample collection at post-mortem.

In contrast, even though the clinical presentation in Outbreak 2 was typical of FCV-VSD with all cases exhibiting head or limb oedema, or jaundice, viruses from these cats had properties predictive of the virulent pathotype in only one of the seven residues. All viruses sequenced from cats in Outbreak 2 with the virulent pathotype were highly conserved, representing a unique FCV strain causing the outbreak. In Outbreak 3, near identical viruses (99.9% nucleotide identity) were isolated from four cats from different households with phenotypes ranging from asymptomatic, to URTD to VSD. The predicted properties for the virulent pathotype were present in four of the seven hypervariable E region amino-acid residue positions. We also analysed several FCV strains isolated from several cats with diarrhoea, of which one had the predicted properties for the virulent pathotype in all seven residues, whilst the others clustered more closely with the typical FCV-URTD pathotype and Outbreak 2 virulent and non-virulent strains on the MCA (Figure 2).

Whilst highly contagious, FCV strains responsible for VSD do not cause clinical disease in all cats, as was seen here in Outbreak 2, and in a previously reported outbreak in the U.S. [7]. Many factors can influence disease phenotype including route of exposure and dose of the infecting virus, as well as age, vaccination and immune status of the host [5]. In addition, there is a spectrum of virulence among FCV strains, rather than two clearly delineated groups of FCV-URTD and FCV-VSD as demonstrated by the identification of non-epizootic forms of FCV-infection from Switzerland, Lichtenstein, the UK and Italy with clinical disease resembling VSD [9,33,34]. Previous investigations to determine a role for co-pathogens are limited to transmission electron microscopy, where only virions with a morphology consistent with FCV were identified [13], or specific PCRs testing for feline parvovirus (FPV), feline herpesvirus-1, feline immunodeficiency virus and feline leukaemia virus [11]. In Italy, three cats from three separate FCV-VSD outbreaks were all co-infected with FPV [11], leading to speculation that FPV-associated immunosuppression may have facilitated systemic spread of low-virulence FCVs or the emergence of virulent FCVs. Concurrent immunosuppression could explain the finding of an FCV-URTD pathotype in Outbreak 2, despite strong evidence of an FCV-VSD phenotype. Future investigations to clarify the potential role of co-pathogens in FCV-VSD are warranted. Other factors implicated in the pathogenesis of VSD include host factors, the presence of viraemia [35], higher viral tissue loads [8] and broader tissue tropism of the virulent pathotype compared to the respiratory pathotype [6,8,9,28,30,33].

FCV was isolated from tissues of cats with FCV-VSD from Outbreak 1 and from oropharyngeal/conjunctival swabs collected prospectively from cats in Outbreaks 2 and 3. Since none of the swab-sampled cats died, it was not possible to culture virus from their tissues. The seven viruses isolated from cats in Outbreak 2 with severe clinical signs characteristic of VSD were identical or near identical and were considered representative of the VSD phenotype.

There is limited information regarding of the efficacy and safety of antiviral compounds against FCV-VSD strains. Accordingly, we investigated the in vitro efficacy of three antiviral compounds (NTZ, 2CMC, NITD-008) against representative strains of FCV-VSD. One isolate (NSW_5_V1) was in a different phylogenetic cluster to the other two (QLD_9 and 12), The NSW isolate had the hypervariable E region residue properties attributed to FCV-VSD pathotypes in the study of Brunet et al. [17], while the QLD isolates had residue properties typical of the FCV-URTD pathotype. These three antiviral molecules showed dose–response inhibition against the replication of FCV-VSD strains at low micromolar concentrations. Nucleoside analogues, 2CMC and NITD-008, inhibit the viral RNA-dependent RNA polymerase. Although the in vitro therapeutic index of 2CMC in our study suggests it would be the safest of the three for treatment of FCV-VSD, clinical development of its 3’-valyl ester oral prodrug valopicitabine was halted in people because of adverse gastrointestinal effects [36]. Similarly, although NITD008 has exhibited in vitro efficacy against human and animal caliciviruses, adverse effects including weight loss, lethargy, nausea and diarrhoea occurred in dogs administered the drug intravenously for 2 weeks [37]. Nitazoxanide (NTZ) is a nitrothiazole benzamide compound approved by the U.S. Food and Drug Administration (FDA) for oral treatment of protozoal infections [38]. Its antiviral activity was discovered serendipitously during treatment of cryptosporidiosis (a parasitic disease) in patients with HIV-AIDS [39]. Since then, NTZ and its active circulating metabolite, tizoxanide, have been shown to have antiviral activity against a broad range of DNA and RNA viruses including hepatitis B, hepatitis C and influenza A virus [40]. By uncoupling oxidative phosphorylation, NTZ and tizoxanide inhibit mitochondrial activity, decreasing cellular ATP and thus inhibiting viral replication. NTZ also targets key viral proteins such as the haemagglutinin of influenza A virus and suppresses the secretion of proinflammatory cytokines [40]. It has both in vitro and in vivo efficacy in cats experimentally infected with FCV-URTD strains, and was well tolerated at dose rates from 5 to 20 mg/kg/day orally [41]. Higher dose rates (75 mg/kg/day) in cats cause vomiting and diarrhoea [42]. Based on our data, NTZ could be considered as a treatment option for FCV-VSD given its in vitro efficacy against the prototype vaccine F9 strain isolated in 1958 and contemporaneous isolates of FCV-VSD. Although the therapeutic index for NTZ (TI = 21) calculated in our study suggests it is selective, randomised controlled study in cats should be performed to further evaluate efficacy and safety in vivo.

## 5. Conclusions

FCV-VSD is a clinical diagnosis that can occur in cats worldwide. The trigger(s) for this severe disease syndrome remains (remain) elusive. Evaluation of the safety and efficacy of nitazoxanide as a specific treatment option for FCV-VSD is warranted.

## Figures and Tables

**Table 1:** Case details of cases of suspected FCV-VSD in Outbreak 1 in New South Wales (NSW) in 2015 and the suspected cases of origin (NSW_1 and NSW_2), disease phenotype, time until first appearance of cytopathic effects (CPEs) in viral culture, duration of illness and outcome.

Case	Date in 2015	Breed, Sex	Age	Origin	Vacc. Status	Exposure History and Clinical Signs	Disease Phenotype	CPEs	Duration of Illness	Outcome
NSW_1 Index case	15/11	DSH F	1.5 m	MCH	Unvacc.	Index case URT signs, lameness.	URTD	N/A	2 d	R
NSW_2 Index case	15/11	DSH F	1.5 m	MCH	Unvacc.	Index case URT signs, lameness.	URTD	N/A	4 d	R
NSW_3	02/12	DSH F	4 m	SCH	Unknown	Pyrexia, lethargy, bilateral forelimb oedema, jaundice. Onset of CS 2 days after a surgical procedure (hindlimb amputation).	VSD	N/A	4 d	D
NSW_4	06/12	DSH M	1.25 m	SCH	Unvacc.	Pyrexia, lethargy, facial and forelimb oedema. Onset of CS 7 days after a surgical procedure (abscess drainage).	VSD	N/A	5 d	D
NSW_5 ^1,2,3^	07/12	DSH FN	6 y	SCH	Incomp.	Pyrexia, multiple limb oedema, jaundice. Onset of CS 4 days after a surgical procedure (abscess drainage).	VSD	24 h	7 d	D
NSW_6	09/12	DSH MN	1 y	SCH	Complete	Pyrexia, forelimb oedema. Onset of CS 4 days after a surgical procedure (abscess drainage).	VSD	N/A	9 d	R
NSW_7	14/12	DSH M	2 y	MCH	Complete	Pyrexia, facial and forelimb oedema, oral ulcers.	VSD	N/A	2 d	E
NSW_8	14/12	DSH FN	3 y	SCH	Complete	Pyrexia, facial oedema. Onset of CS 5 days after a surgical procedure (jaw fracture repair).	VSD	N/A	1 d	E
NSW_9 ^1,2^	18/12	DSH F	1.5 m	MCH	Unknown	Pyrexia, facial oedema. Onset of CS 4 days after a surgical procedure (for intestinal intussusception).	VSD	24 h	1 d	E

^1^ Samples collected for viral culture. ^2^ Whole-genome sequencing performed. ^3^ Isolate NSW_5_V1 was tested against 3 antiviral agents in vitro (Figure 4). CS, clinical signs; d, days; D, died; DSH, domestic shorthair; E, euthanised; F, female; FN, female neutered; Incomp., incomplete; m, months; M, male; MN, male neutered; MCH, multicat household; N/A, not applicable; R, recovered; SCH, single-cat household; Unvacc., unvaccinated; URTD, upper respiratory tract disease; Vacc., vaccinated; VSD, virulent systemic disease; y, years.

**Table 2:** Case details of cases of suspected FCV-VSD in Outbreak 2 in Queensland (QLD) in 2017 and the suspected cases of origin (NSW_1 and NSW_2), disease phenotype, time until first appearance of cytopathic effects (CPEs) in viral culture, duration of illness and outcome.

Case	Date in 2017	Breed, Sex	Age	Origin	Vacc. Status	Exposure History and Clinical Signs (CS)	Disease Phenotype	CPEs	Duration of Illness	Outcome
QLD_1	28/08	DSH FN	3 y	MCH	Complete	Acute respiratory effort, jaundice. Onset of CS 5 days after a surgical procedure (neutering).	VSD	N/A	2 d	D
QLD_2	30/08	DSH FN	1 y	SCH	Complete	URT signs, facial oedema, oral ulcers. Onset of CS 5 days after a surgical procedure (neutering).	VSD	N/A	9 d	R
QLD_3	06/09	DSH MN	4 y	SCH	Complete	Pyrexia, jaundice, facial/limb oedema. Onset of CS 7 days after a surgical procedure (abscess drainage).	VSD	N/A	13 d	R
QLD_4	25/9	DSH FN	10 m	SCH	Complete	Pyrexia, facial/limb oedema, jaundice, dyspnoea. Onset of CS 4 days after a surgical procedure (neutering).	VSD	N/A	6 d	E
QLD_5 *^,1,2^	05/10	DSH MN	1 y	MCH	Complete	Pyrexia, facial/limb oedema, jaundice, oral/skin ulcers. Onset of CS 9 days after a surgical procedure (limb amputation).	VSD	48 h	6 d	R
QLD_6 **^,1,2^	06/10	DSH FN	1 y	MCH	Complete	Lameness, limb oedema, oral ulcers, elevated bilirubin. Onset of CS 7 days after a surgical procedure (neutering).	VSD	48 h	6 d	R
QLD_7 **^,1^	09/10	DSH FN	1 y	MCH	Complete	Pyrexia, inappetence, limb oedema, elevated bilirubin. Onset of CS 10 days after a surgical procedure (neutering).	VSD	72 h	10 d	R
QLD_8 *^,1,2^	09/10	DSH MN	2 y	MCH	Complete	Pyrexia, inappetence, facia/limb oedema, oral ulcers, nasal discharge, sneezing. Household contact of QLD_5.	VSD	48 h	7 d	R
QLD_9 ***^,1,2,3^	09/10	DSH FN	1 y	MCH	Complete	Pyrexia, anorexia, limb oedema, nasal discharge, dyspnoea, jaundice. Onset of CS 11 days after a surgical procedure (neutering).	VSD	36 h	8 d	R
QLD_10 ***^,1,2^	09/10	DSH FN	1 y	MCH	Complete	Pyrexia, nasal discharge, facial/limb oedema. Onset of CS 11 days after a surgical procedure (neutering).	VSD	36 h	6 d	R
QLD_11 ***^,1^	09/10	DSH FN	3 y	MCH	Complete	Pyrexia, facial/limb oedema, jaundice, nasal discharge, hypothermia. Household contact of QLD_9 and QLD_10.	VSD	72 h	6 d	E
QLD_12 ^1,2,3^	03/10	DSH FN	6 y	MCH	Complete	Pyrexia, anorexia, limb oedema, elevated bilirubin. Onset of CS 5 days after treatment for brown snake envenomation.	VSD	36 h	4 d	R
QLD_13 ^1,2^	15/10	Unkn.	N/A	MCH	Unkn.	Adult stray cat from a colony, oral ulcers.	URTD	N/A	Unkn.	Unkn.

* Same household; ** same household; *** same household. ^1^ Samples collected for viral culture. ^2^ Whole-genome sequencing performed. ^3^ Isolates QLD_9 and QLD-12 were tested against 3 antiviral agents in vitro (Figure 4). CS, clinical signs; D, died; DSH, domestic shorthair; E euthanised; F, female; FN, female neutered; Incomp., incomplete; m, months; M, male; MN, male neutered; MCH, multicat household; N/A, not applicable; R, recovered; SCH, single-cat household; Unkn., unknown; Unvacc., unvaccinated; URTD, upper respiratory tract disease; Vacc., vaccinated; VSD, virulent systemic disease; y, years.

**Table 3:** Case details of cases of suspected FCV-VSD in Outbreak 3 in the Australian Capital Territory (ACT) in 2018 and the suspected case of origin, disease phenotype, time until first appearance of cytopathic effects (CPEs) in viral culture, duration of illness and outcome.

Case	Date in 2018	Breed, Sex	Age	Origin	Vacc. Status	Exposure History and Clinical Signs (CS)	Disease Phenotype	CPEs	Duration of Illness	Outcome
ACT_1 **^,1,2^ Index case	22/01	DSH FN	4 m	MCH	Incomp.	Fever, inappetence, lameness, polyarthropathy. Adopted from a shelter.	URTD	36 h	14 d	R
ACT_2 **^,1,2^ Index case	22/01	DSH MN	4 m	MCH	Incomp.	Fever, lethargy, sneezing. Adopted from a shelter.	URTD	36 h	14 d	R
ACT_3 ****^,1,2^	23/01	DSH M	10 y	MCH	Unvacc.	No CS. Adopted from a rescue society. Presented on 23/01 for a dental procedure.	Asympt.	60 h	N/A	N/A
ACT_4 ****	30/01	DSH FN	5 y	MCH	Complete	Fever, pain on abdominal palpation, limb oedema, myopathy, creatine kinase 20,206 U/L (RR < 261); AST 487 U/L, (RR < 60), myoglobinuria. Indoor cat co-housed with ACT_3. Onset of CS 7 days after ACT_3 had a dental procedure.	VSD	N/A	9 d	R
ACT_5	02/02	DSH MN	8 y	MCH	Complete	Fever, inappetence, hypersalivation, jaundice, lumbar muscle pain, facial/limb oedema, ulcerated nasal planum. Onset of CS 4 days after a dental procedure.	VSD	N/A	21 d	R
ACT_6 ***	07/02	DSH FN	5 y	MCH	Complete	Vomiting, fever, painful kidneys on abdominal palpation, marked facial and limb oedema (all limbs), subcutaneous oedema of flanks, oral ulcers, swollen nose. Onset of CS 14 days after dental check-up.	VSD	N/A	10 d	R
ACT_7 *^,1,2^	08/02	Maine Coon MN	10 m	MCH	Complete	Fever, inappetence, oral ulcers. Onset of CS 8 days after ACT_8 had a dental procedure.	URTD	48 h	7 d	R
ACT_8 *	08/02	DSH MN	15 y	MCH	Unknown	Fever, inappetence, nasal planum ulcers. Onset of CS 8 days after being admitted to the hospital for a dental procedure.	URTD		12 d	R
ACT_9 ***^,1,2^	14/02	Ragdoll cross MN	4 y	MCH	Comp.	Fever, inappetence, lethargy, facial/limb oedema, swollen nose. Indoor cat co-housed with ACT-6.	VSD	36 h	7 d	R

* From same household; ** from same household; *** from same household. **** from same household ^1^ Samples collected for viral culture. ^2^ Whole-genome sequencing performed. Comp., complete; CS, clinical signs; D, died; DSH, domestic shorthair; E, euthanised; F, female; FN, female neutered; Incomp., incomplete; M, male; MN, male neutered; MCH, multicat household; N/A, not applicable; R, recovered; SCH, single-cat household; Unvacc., unvaccinated; URTD, upper respiratory tract disease; Vacc., vaccinated; VSD, virulent systemic disease; wks, weeks; y, years.

**Table 4:** Amino-acid residues from the E region of feline caliciviruses including regions with high variability 426–460 (N-HV portion) and 490–523 (C-HV portion), separated by a less-variable region (aa 461–489) (Brunet et al., 2019).

Strain	Amino-Acid Residues and Physico-Chemical Properties Associated with VSD Pathotype						
	438 Hydrophobic, Aliphatic ILV	440 Non-Small EFHKIL MQRWY	448 Polar, Positive Charge HKR	452 Non-Small EFHKIL MQRWY	455 Non-Negative All Except DE	465 Polar DEHKN QRSTWI	492 Small ADGNP STV
FCV-VSD	V_9_T_7_ (9)	Q_6_G_4_E_4_SK (11)	K_7_A_2_E_2_7 (7)	E_11_D_6_ (11)	T_6_D_3_M_2_I_2_NES (12)	S_14_G_3_ (14)	V_16_R (16)
FCV-URTD	T_37_ V_2_I	G_22_S_6_Q_4_R_2_A_2_END	A_30_P_4_G_3_K_3_	D_36_E_3_ N	D_28_T_5_S_3_G_2_VR	G_26_S_14_	V_17_L_8_I_6_ R_5_K_2_
E-ITA/2013/160	V	Q	R	E	T	G	V
E-FCV ITA others	T_3_ V_11_	G_2_SR	A_4_	D_4_	D_3_E	G_3_S	L_3_V_2_
E-ITA/201X/81	V	S	A	D	E	S	V
E-ITA/201X/I48	T	R	A	D	D	G	L
E-ITA/201X/I80	T	G	A	D	D	G	L
E-ITA/201X/I82	T	G	A	D	D	G	L
ACT7_URTD	T	G	**K**	**E**	D	**S**	**V**
ACT9_VSD	T	G	**K**	**E**	D	**S**	**V**
ACT1_URTD	T	G	**K**	**E**	D	**S**	**V**
ACT3_Asympt.	T	G	**K**	**E**	D	**S**	**V**
ACT2_URTD	T	G	**R**	**E**	**N**	**S**	I
QLD13_URTD	T	G	A	D	D	G	**V**
QLD8_VSD	T	G	A	D	D	G	**V**
QLD 5_VSD	T	G	A	D	D	G	**V**
QLD6_VSD	T	G	A	D	D	G	**V**
QLD9_VSD	T	G	A	D	D	G	**V**
QLD10_VSD	T	G	A	D	D	G	**V**
QLD12_VSD	T	G	A	D	D	G	**V**
NSW5_V1_VSD	**V**	**Q**	**K**	**E**	**I**	**S**	**V**
NSW9_V1_VSD	**V**	**Q**	**K**	**E**	**I**	**S**	**V**
NSW5_V2_VSD	T	S	**K**	**E**	D	**S**	**V**
NSW9_V2_VSD	T	S	**K**	**E**	D	**S**	**V**

A (blue): hydrophobic small; V (blue): hydrophobic aliphatic small; I (blue): hydrophobic aliphatic; M (blue): hydrophobic. T (green): hydrophobic polar small; S (green): polar small; Q (green): polar. D (pink): polar small charge -ve; E (pink): polar charge -ve. K (red): hydrophobic polar charge +ve; R (red): polar charge +ve. G: hydrophobic small. VSD, virulent strains; R, respiratory strains; E-ITA, enteric strains from cats with diarrhoea. Grey-shaded cells in the table with bolded font correspond to amino-acids of strains in this study with the predicted properties of VSD strains according to Brunet et al., 2019 [17].

## Data Availability

The sequence data analysed in this project are publicly available on the BioProject database (Submission ID SUBID PRJNA768617 at https://www.ncbi.nlm.nih.gov/bioproject/768617, accessed on 12 August 2021).

## Supplementary Materials

The following are available online at https://www.mdpi.com/article/10.3390/v13102040/s1, Table S1: Summary of RNA-sequencing results of RNA extracted from oropharyngeal/conjunctival swabs or tissues from sampled cats in this study.
