# Supplementary material for: Feline Calicivirus Virulent Systemic Disease: Clinical Epidemiology, Analysis of Viral Isolates and In Vitro Efficacy of Novel Antivirals in Australian Outbreaks
Source: Viruses. 2021 Oct 9;13(10):2040. doi: 10.3390/v13102040 (PMC8537534; doi:10.3390/v13102040)
Supplement: Supplementary file 1 [file viruses-13-02040-s001.zip › viruses-1362101-supplementary.pdf]

viruses

MDPI

Supplementary Table S1: Summary of RNA-sequencing results of RNA extracted from oropharyngeal/conjunctival swabs or tissues from sampled cats in this study.

Cases	GenBank Accession No.	Read Count	Contigs	Reads Mapped	Reads per Million
NSW5_V1	MW880757	127241260	30735	106347262	835792.27
NSW5_V2	MW880758	112946412	891879	152719	1352.14
NSW9_V1*	-	131951868	24750	99536924	754342.67
NSW9_V2	MW880759	131951868	24750	8569729	64945.87
QLD5	MW880766	126373048	141343	15854083	125454.62
QLD6	MW880765	142583738	101261	18998254	133242.78
QLD8	MW880771	122917554	28307	22086249	179683.44
QLD9	MW880761	123342872	163172	14335903	116228.06
QLD10	MW880762	125677722	20343	19605713	155999.91
QLD12	MW880760	125046816	98305	16353907	130782.27
QLD13	MW880763	83299626	1860	42785903	513638.60
ACT1	MW880767	142608064	73633	47644293	334092.56
ACT2	MW880769	134000790	33540	107954102	805622.88
ACT3	MW880764	94284000	5430	43940299	466041.95
ACT7	MW880768	145687464	18221	52198716	358292.43
ACT9	MW880770	117711790	17228	42174952	358289.96
**Total**	-	1855673024	1650007	1516128084	817023.29
**Median**	-	126025385	32137	32130600	254953.40

* Identical sequence to NSW5_V1
